# Frequency Schrödinger bridge diffusion model for fast MRI reconstruction in abdominal MR‐guided online adaptive radiotherapy

**DOI:** 10.1002/acm2.70831

**Published:** 2026-10-09

**Authors:** Zhiqun Wang, Shaobin Wang, Nan Liu, Qiu Guan, Qi Chen, Fang Chen, Bei Wang, Yongguang Liang, Xia Liu, Bo Yang, Lang Yu, Hongen Liao, Jie Qiu

**Affiliations:** ^1^ Department of Radiation Oncology Chinese Academy of Medical Sciences Peking Union Medical College Beijing China; ^2^ School of Biomedical Engineering Tsinghua University Beijing China; ^3^ MedMind Technology Co., Ltd Beijing China; ^4^ School of Biomedical Engineering and the Institute of Medical Robotics Shanghai Jiao Tong University Shanghai China

**Keywords:** abdominal imaging, fast MRI reconstruction, frequency Schrödinger bridge diffusion (FSBD), MR‐guided online adaptive radiotherapy

## Abstract

**Background:**

Online adaptive radiotherapy (ART) employs modalities like CBCT, CT, and MRI. For abdominal malignancies, MRI stands out with superior soft‐tissue contrast, no ionizing radiation, and functional imaging capabilities. Yet, MRI‐guided ART for such cases is challenged by long treatment time. Thus, boosting efficiency, especially via accelerated MRI scanning, is vital.

**Purpose:**

This study presents the first accurate and fast MRI reconstruction method tailored for the Unity MR‐Linear system. By slashing MRI acquisition time, it aims to enhance patient compliance, reduce intra‐fraction anatomical motion, and optimize clinical resource use.

**Methods:**

A frequency Schrödinger bridge diffusion (FSBD) model for MRI reconstruction (converting 22‐second MRI to full‐duration MRI) was proposed, based on a retrospective analysis of 31 fractional MRI scans from 14 patients. Its reconstruction performance was compared with state‐of‐the‐art methods: paired diffusion‐based Refusion, and unpaired CycleGAN and CSGAN. Of the initially acquired 40 fractional scans, 9 examinations were excluded following predefined exclusion criteria, leaving 31 valid cases. Dataset was split strictly at patient level: 23 fractional scans for training and the remaining independent patients for testing. An extra baseline using pairwise full‐duration MRI from different fractions of identical patients was supplemented for reliability verification. All metrics were calculated on complete 3D volumetric MRI rather than individual 2D slices.

**Results:**

Quantitative evaluation shows the FSBD model's superiority. It achieved significantly lower error metrics: MAE (86.00) and RMSE (123.42), outperforming Refusion (MAE: 99.95, RMSE: 172.55), CycleGAN (MAE: 112.92, RMSE: 149.16), and CSGAN (MAE: 103.04, RMSE: 162.29). In image quality, FSBD's PSNR (28.44 dB) and SSIM (0.885) surpassed Refusion (26.77 dB / 0.870), CycleGAN (25.82 dB / 0.881), and CSGAN (26.34 dB / 0.878). Moreover, it delivered high‐fidelity reconstruction with an efficient 20‐s inference time.

**Conclusions:**

This study is the first to propose a sub‐one‐minute MRI imaging method for the Unity MR linear accelerator in an MR‐guided online adaptive radiotherapy (ART) workflow. Experimental results confirm the method's significantly better reconstruction performance than state‐of‐the‐art approaches. Total MRI imaging time dropped drastically from 3 min (full‐duration) to 42 s (22 seconds for MR imaging plus 20 seconds for reconstruction). This reduction brings notable clinical benefits, including improved patient compliance and fewer intra‐fractional anatomical variations.

## 1 INTRODUCTION

Online adaptive radiotherapy (ART), a cornerstone of precision radiation oncology, has been widely implemented across multiple imaging modalities, including cone‐beam computed tomography (CBCT), computed tomography (CT), and magnetic resonance imaging (MRI). Studies demonstrate that CBCT‐guided online ART significantly reduces planning target volume (PTV) margins to 5 mm in postoperative gynecologic cancers, minimizing acute toxicity while maintaining target coverage.^1^ Meanwhile, hybrid CT‐MR imaging enhance dynamic plan adaptation by leveraging complementary anatomical information.^2^ Among these modalities, MRI has emerged as a transformative tool due to its unparalleled advantages: superior soft‐tissue contrast enables precise delineation of abdominal and pelvic targets (e.g., bladder, rectum) and organs at risk (OARs);^3^, ^4^ absence of ionizing radiation permits repeated imaging without cumulative dose concerns;^5^ and functional imaging capabilities, such as diffusion‐weighted imaging, provide biological insights for dose painting.^6^ Furthermore, real‐time MRI monitoring of respiratory motion, combined with motion management techniques (e.g., gating, abdominal compression), enhances targeting accuracy for abdominal malignancies, including pancreatic and hepatic tumors.^4^, ^7^

Current clinical implementations of MRI‐guided ART for abdominal malignancies highlight both promise and challenges. For instance, 1.5T MR‐Linac systems have enabled dose escalation (> 70 Gy biologically effective dose) in pancreatic cancer with reduced intestinal exposure through daily adaptive replanning.^4^, ^7^ However, prolonged treatment duration remains a critical clinical challenge: a single fraction can take 55–79 min.^4^ Efficiency improvements are required at every technical step, with MRI scanning time being a current priority for optimization. Accelerating scans is crucial to enhance patient compliance, minimize intra‐fractional anatomical variations, and reduce staffing resource demands. Studies have reported that replacing conventional T2‐weighted imaging (360 s) with optimized T1‐weighted protocols (120 s), have mitigated this issue while preserving image quality for contouring accuracy.^7^ Such efficiency gains improve patient compliance, mitigate intra‐fractional anatomical shifts, and reduce clinical resource burdens.^5^, ^6^, ^8^

Shortening the MR imaging time can improve patient comfort and compliance. A long MR examination (3 minutes) has extremely high requirements for patients to maintain their body position. Especially for children, elderly patients, patients with pain, or those with claustrophobia, it is easy to cause irritability, movement, and even failure of the examination. Reducing the MR imaging time to 1 min can significantly reduce patients' discomfort, reduce image artifacts caused by body position movement, and improve the success rate of the examination. In addition, shortening the MR imaging time can improve the efficiency of equipment utilization and lay a time foundation for precise radiotherapy. MR is the “gold standard” for delineating target areas (such as the prostate and brain tumors) and organs at risk due to its excellent soft tissue contrast. However, the traditional 3‐min imaging time has limited its application in “real‐time/online adjustment” scenarios. Reducing the MR imaging time to 1 min can provide key time support for MR‐guided precise radiotherapy (such as online adaptive radiotherapy).

The process of online adaptive radiotherapy is as follows: patient positioning before treatment → real‐time image acquisition (to confirm the position of the target area/organs at risk) → comparison with the planned images → rapid adjustment of the radiotherapy plan → implementation of treatment. The entire process needs to be completed on the day of treatment, and the total time must be controlled within a range that patients can tolerate (usually ≤ 30 minutes); otherwise, it will be difficult for patients to maintain their body position, resulting in low treatment efficiency. Among them, the 3‐minute MR imaging time was once a major obstacle to online adaptive radiotherapy because excessively long image acquisition will prolong the total treatment time, leading to patient body position movement or treatment delay. If the MR imaging time is reduced to 1 min, combined with the following technological advancements, it can significantly promote the realization of Online ART: currently, AI‐assisted image registration (such as deformation registration based on deep learning) and dose plan optimization (such as fast Monte Carlo algorithm) can be completed within a few minutes. Combined with 1‐minute imaging, the total time of the entire Online ART process can be controlled within 20–30 min (a range tolerable to patients). In addition, MR‐Linac (magnetic resonance‐guided linear accelerator) can perform real‐time imaging during treatment, and 1‐min MR imaging can be more efficiently linked with treatment equipment to achieve seamless connection of “imaging—adjustment—treatment”.

The primary strategy for accelerated MRI scanning involves undersampling k‐space data and then reconstructing images based on prior knowledge derived from both the image and measurements, such as parallel imaging and compressed sensing. In recent years, deep learning (DL)‐based reconstruction methods have gained increasing popularity and demonstrated significant potential in eliminating aliasing artifacts caused by undersampling.^9^ Generative Adversarial Network (GAN) is used in^10^ to estimate missing k‐space samples. By introducing adversarial loss through adversarial interaction between the generator and the discriminator, brain MRI reconstruction can be achieved. An MRI super‐resolution reconstruction method based on scan‐specific generative neural network is proposed in,^11^ using a regression network based on the 3D UNet architecture. The 3D UNet architecture is also utilized in^12^ to achieve fast surface reconstruction of the neonatal cortex. For MRI reconstruction,^13^ proposes a cascaded pyramid convolutional RNN containing three convolutional recurrent neural networks. To recover fully‐sampled MRI data from under‐sampled k‐space data,^14^ develops a self‐supervised adaptive diffusion model.^15^ presents a fast, calibration‐free low‐rank parallel MRI reconstruction method based on unfolded deep learning to estimate multi‐channel spatial support maps. Different from previous methods, we are the first to investigate a fast MRI imaging acceleration optimization method for the Unity MR linear accelerator.

For DL‐based image translation task, GAN^16^ and CycleGAN^17^ are widely used.^18^, ^19^ Recently, score‐based generative models (SGMs, also known as diffusion models), have emerged as a pivotal research direction in image generation.^20^, ^21^ While these models demonstrate remarkable performance in tasks such as image synthesis, they exhibit several critical limitations: First, significant efficiency bottlenecks persist. The requirement to discretize continuous‐time dynamics typically necessitates hundreds to thousands of timesteps for distribution mapping, resulting in computationally intensive iterative sampling that severely hinders real‐time applications. Second, noise scheduling strategies remain heavily dependent on empirical heuristics. Existing methods predominantly determine timestep‐specific noise parameters (e.g., variance scaling coefficients) through trial‐and‐error approaches, lacking theoretically grounded adaptive optimization mechanisms. Third, models exhibit limited capacity to capture low‐dimensional manifolds in high‐dimensional data. When handling tasks involving structured high‐dimensional data, the inherent Gaussian prior assumption restricts their ability to model complex spatiotemporal dependencies. Finally, the unidirectional nature of generation constrains application scope. Conventional diffusion models only support unidirectional mapping from simple priors, such as Gaussian distributions, to target data distributions. This limitation precludes bidirectional transformations between arbitrary complex distributions.

To achieve accurate MRI reconstruction, we introduce a novel Frequency‐domain Schrödinger Bridge Diffusion (FSBD) model. The Schrödinger Bridge framework identifies an optimal stochastic path connecting two probability distributions,^22^, ^23^ thereby enhancing traditional diffusion models with optimal transport theory. This formulation guides the model to focus on local regions along the most probable transformation path. Specifically, our FSBD model builds upon the unpaired neural Schrödinger bridge. This foundation enables unpaired image translation between datasets, providing an efficient probabilistic pathway for handling misaligned source and target images. We frame the Schrödinger Bridge problem as an adversarial learning task, utilizing discriminators and regularization to learn the transformation bridge for MRI reconstruction. Concretely, the approach minimizes transportation cost under Kullback‐Leibler (KL) divergence constraints. It employs a Markov chain to model distribution paths and utilizes generator networks within the adversarial learning framework to approximate the forward and backward bridge processes. Furthermore, we introduce a Fourier convolution module for frequency‐domain processing. By complementing existing spatial‐domain convolution feature learning with Fourier‐based frequency‐domain feature extraction, the FSBD model significantly enhances its discriminative feature learning capability, leading to a substantial boost in MRI reconstruction accuracy.

By using the proposed fast MRI reconstruction method, the total MRI imaging time is significantly reduced from 3 min to 42 s. Overall, this study demonstrates the pioneering deployment of sub‐one‐minute rapid MR imaging (≤60 seconds) within an MR‐guided online adaptive radiotherapy (ART) workflow, resulting in a scan time reduction of over 80% compared to conventional MR sequences. In this way, the radiation therapy plan can be adjusted in a timely manner, which significantly enhances the patient's experience during treatment. Meanwhile, the intra‐fractional anatomical movements can be significantly reduced.

## 2 MATERIALS AND METHODS

### 2.1 Patient characteristics

This study included 14 abdominal (liver/pancreas) cancer patients (9 males, 5 females) treated with a 1.5 T Elekta Unity MR‐Linac at our institution. The cohort had a mean age of 63.6 ± 14.2 years (range: 30–84 years), with treatments administered between October 2024 and January 2025. A total of 40 fractional scans were originally collected. Nine scans were excluded due to severe motion artifacts, incomplete scan sequences and poor image definition per predefined exclusion criteria.

All enrolled patients received standardized abdominal compression before scanning. Respiratory motion was monitored via MRI‐Cine sequences, with tumor target motion controlled below 5 mm to reduce respiratory‐induced anatomical mismatch between sequential scans.

### 2.2 MRI acquisition protocol

All scans were performed using a standardized T2‐weighted 3D turbo spin‐echo (T2 3D Tra) sequence with the following parameters: voxel size = 1.5 × 1.5 × 5.00 mm^3^, echo time (TE) = 268 ms, repetition time (TR) = 2100 ms, and field of view (FOV) = 300 mm (AP, frequency) × 403 mm (RL, phase) × 200 mm (FH). The acquisition matrix was 200 × 268 × 80 slices with a default slice gap of ‐2.5 mm, supplemented by spectral presaturation with inversion recovery (SPIR) for fat suppression.

To optimize the signal‐to‐noise ratio (SNR), the number of signal averages (NSA) was set to 5. The standard scan duration for this sequence was 3 min and 22 sec (3:22). For model training, accelerated scans with duration of 22 s (NSA = 1) were acquired as input data, while the corresponding full‐duration (3:22) standard scans served as reference images for ground truth comparison.

### 2.3 Method

Online adaptive radiotherapy (ART) leverages imaging modalities such as cone‐beam computed tomography (CBCT), computed tomography (CT), and magnetic resonance imaging (MRI). Among these, MRI stands out due to its superior soft‐tissue contrast, non‐ionizing radiation nature, and capabilities for functional imaging, which significantly contribute to the advancement of abdominal cancer treatment. Nevertheless, the implementation of MRI‐guided ART for abdominal malignancies is fraught with difficulties, with the protracted treatment durations being a primary concern. Enhancing the overall efficiency, particularly the speed of MRI scanning, has thus become a pressing imperative. In this study, we aim to explore and develop an accurate and rapid MRI reconstruction technique for the Unity MR linear accelerator. As shown in Figure 1, we propose a fast MRI reconstruction model (20 seconds) on the basis of 22S‐duration MRI. In this way, the processing time of the whole pipeline is 42 s, significantly reducing the imaging time of traditional full‐duration MRI (∼3 minutes).

**FIGURE 1:**
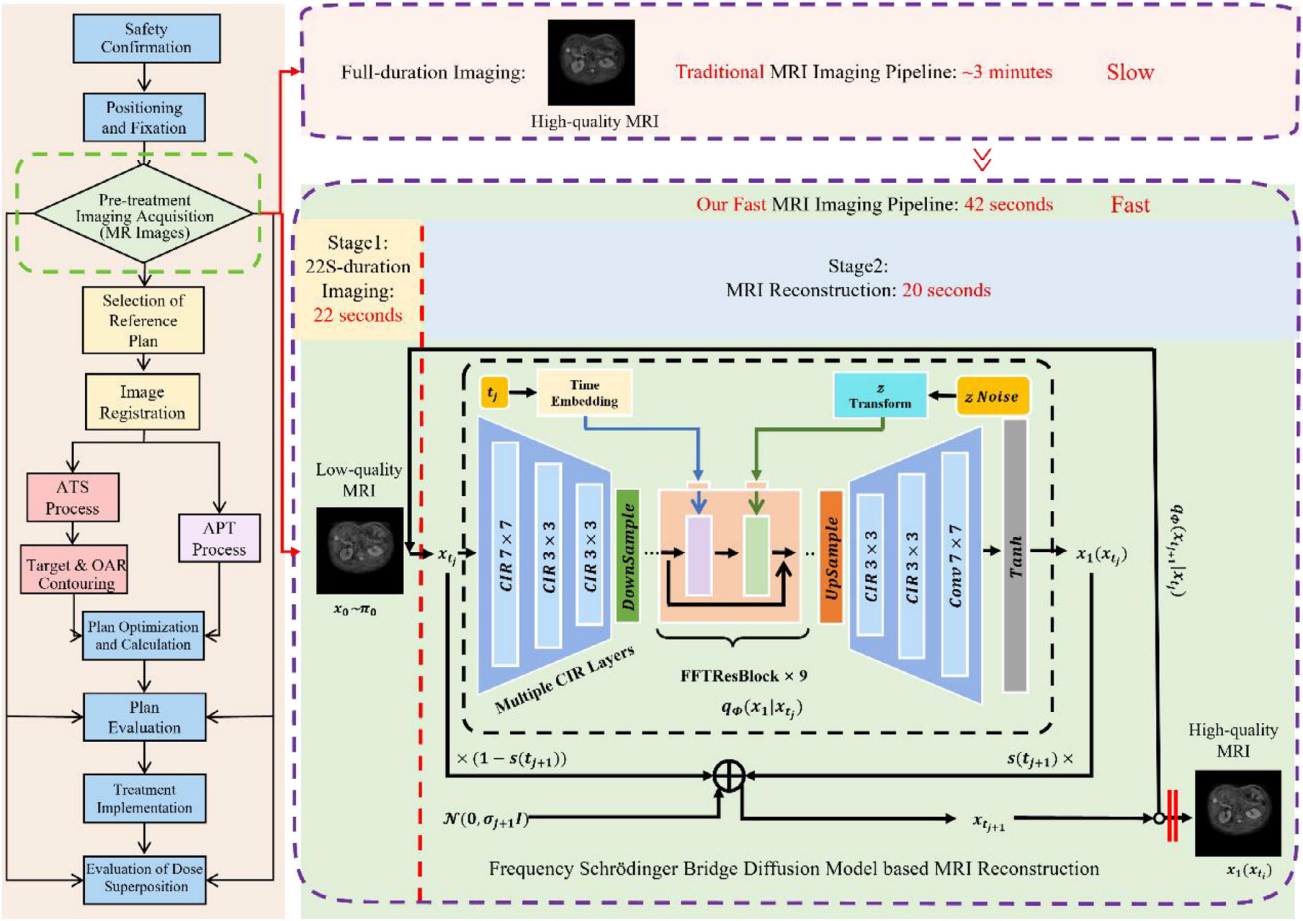
The proposed MRI reconstruction pipeline dramatically reduces the imaging time from ∼3 minutes to 42 s.

To achieve high‐precision MRI reconstruction, this study proposes a frequency Schrödinger bridge diffusion (FSBD) model based reconstruction method. The Schrödinger bridge problem attempts to address the following issue: given two boundary probability distributions $p_{0}$ and $p_{1}$, find a stochastic path (described as a jointly probability distribution $P(x_{t})$ evolving over time) that can optimally connect two distributions. On this basis, the traditional diffusion model is improved with the modeling mode of finding the optimal transport, which effectively guides the model to focus on local areas along the path of the correct transformation.

Diffusion models are a class of generative models. Their core idea is to transform the data distribution into a simple distribution (such as a Gaussian distribution) by gradually introducing noise. Subsequently, the noise is restored back to the data distribution by learning the inverse process. Diffusion models based on deep neural networks learn the process from transforming from a Gaussian distribution to a specific complex distribution, making them suitable for image generation tasks. However, the original diffusion models are limited to generating images of a specific distribution from a Gaussian distribution and are not applicable to the image translation task of MRI reconstruction.

Diffusion models use the Gaussian prior assumption, which leads to limitations in image‐to‐image translation tasks. Schrödinger bridge learns to transform between two arbitrary distributions of stochastic differential equations and can be used to solve the problem of image translation. Specifically, the Unpaired Neural (UN) Schrödinger bridge can successfully perform unpaired image translation tasks between two image sets, providing an efficient probabilistic path framework for handling misaligned data between the source image set and the target image set. Our model formulates the Schrödinger bridge problem as a series of adversarial learning problems, and combines advanced discriminators and regularization method to learn the UN Schrödinger bridge between unpaired image data, which can be used for the reconstruction of MRI.

The method of the UN Schrödinger bridge for handling the Schrödinger bridge problem is shown in Figure 2. Here $q_{\Phi }$ represents the neural network for diffusion used in image translation. This method uses a neural network to implement the Schrödinger Bridge process and trains the model by minimizing the Schrödinger bridge loss. The Schrödinger bridge loss can be expressed as:

(1)

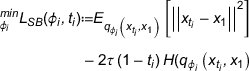




**FIGURE 2:**
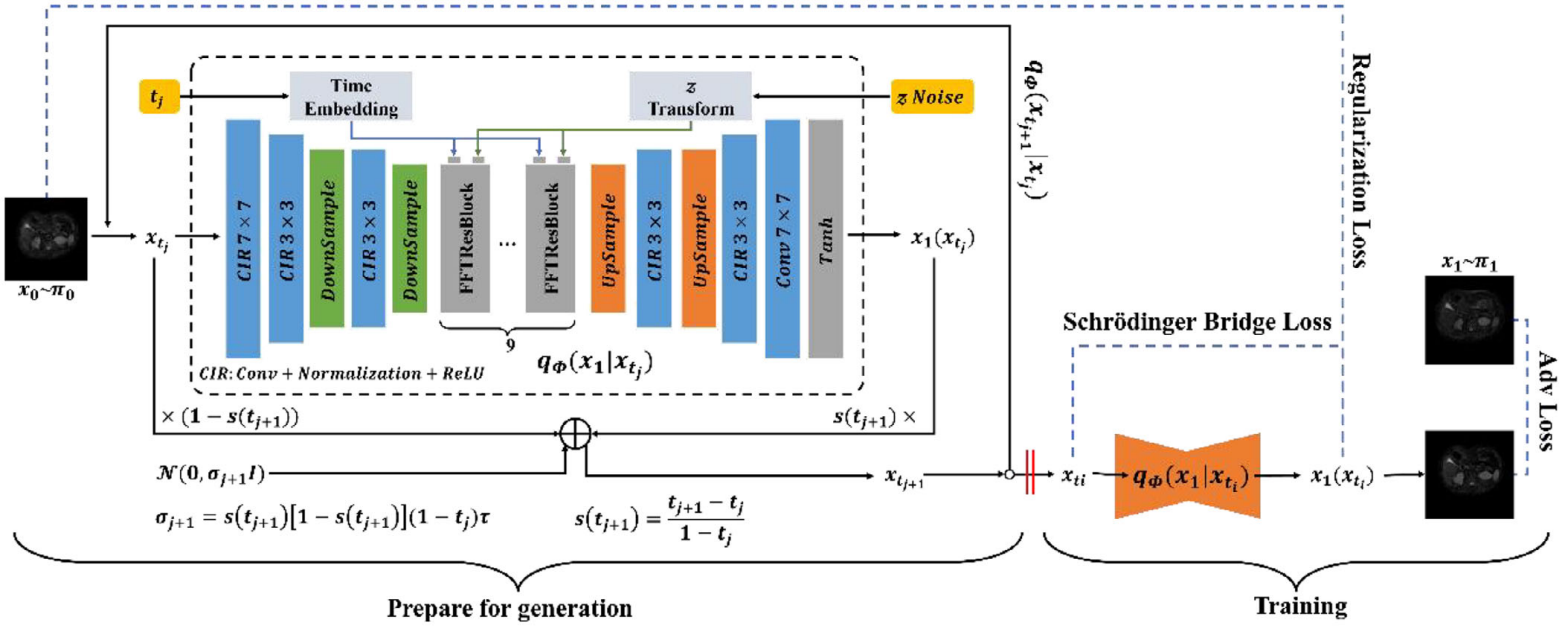
Details of the proposed frequency Schrödinger bridge diffusion (FSBD) model for MRI reconstruction.

Among them, $x_{t_{i}}$ represents the noisy image produced by the diffusion model, which is mathematically defined by the formula in the dashed box. $x_{1}$ represents the generated fake image, that is, $x_{1}\left(x_{t_{i}}\right)$ in the Figure 2. $E_{q_{\phi_{i}}\left(x_{t_{i}},x_{i}\right)}\left[∥x_{t_{i}}-x_{i}{∥}^{2}\right]$ is the mean value of the distance between the two, which is used to make the diffusion process tend to optimal transport. $q_{\phi_{i}}\left(x_{t_{i}},x_{1}\right)$ can be expressed as:

(2) $$q_{\phi_{i}}\left(x_{t_{i}},x_{1}\right)≔q_{\phi_{i}}\left(x_{1}|x_{t_{i}}\right)p\left(x_{t_{i}}\right)$$

(3) $$q_{\phi_{i}}\left(x_{1}\right)≔E_{p\left(x_{t_{i}}\right)}\left[q_{\phi_{i}}\left(x_{1}|x_{t_{i}}\right)\right]$$

That is, $q_{\phi_{i}}\left(x_{t_{i}},x_{1}\right)$ denotes the joint distribution of the generated fake image and the conditional distribution, and $q_{\phi_{i}}\left(x_{1}\right)$ represents the distribution of the generated fake image. The discriminator estimates  the probability that the fake image belongs to the target domain. The latter term of the Schrödinger bridge loss represents the information entropy of the probability of the fake image, and the entropy can be inferred indirectly from the relationship between cross‐entropy and Kullback‐Leibler (KL) divergence, since the cross‐entropy is equal to the entropy plus the KL divergence.

Our method performs the diffusion process in the intermediate feature space rather than directly on the image. It leverages the characteristics of deep features while reducing computational burden and improving semantic alignment across time steps. Specifically, we utilize a bidirectional stochastic framework, consisting of a forward generator $q_{\phi_{f}}\left(x_{1}|x_{t_{i}}\right)$ and a backward generator $q_{\phi_{b}}(x_{t_{i}}|x_{1}$) both parameterized by neural networks. The network consists of multiple residual blocks, with features obtained from the intermediate blocks contributing to the diffusion process. During training, a noisy latent variable $x_{t_{i}}$ is generated at a randomly sampled time step $t_{i}$ using a predefined noise schedule applied to intermediate features. The forward generator learns to predict the clean representation $x_{1}$, while the backward generator reconstructs $x_{t_{i}}$ from $x_{1}$, forming a time‐symmetric stochastic process. This dual generator framework is optimized using adversarial training objectives, combined with a bidirectional consistency constraint that ensures path coherence between the forward and backward trajectories in feature space. Together, this design enforces smooth transitions across diffusion steps and enhances the reconstruction fidelity of the model.

In addition, we adopt a Fourier convolution frequency domain processing module. This module augments the existing spatial domain convolution feature learning with Fourier convolution frequency domain feature learning. By doing so, it enhances the network model's ability to learn discriminative features, thereby further improving the accuracy of MRI reconstruction.

Fourier convolution applies convolution operations to the feature map in the Fourier domain. After applying the Fourier transform and before performing the inverse transform back to the spatial domain, this approach facilitates the encoding of detailed information from different frequency domain components of the image, thereby enhancing feature extraction during the reconstruction process. Specifically, we present a diffusion Fourier residual generative network, and the structure of the generative model $q_{\phi }$ describing the evolution of the Schrödinger Bridge distribution path is shown in Figure 3. In the proposed architecture, the input feature maps are first passed through a 2D Fourier transform (FFT) to convert spatial features into frequency domain representations. These frequency features are then separated into real and imaginary components and processed using two parallel convolutional branches with shared or synchronized parameters. After convolution, the real and imaginary components are recombined into complex form, followed by an inverse Fourier transform (IFFT) to map features back to the spatial domain. A skip connection from the input feature is added to the output of the IFFT operation to form the Fourier residual block.

**FIGURE 3:**
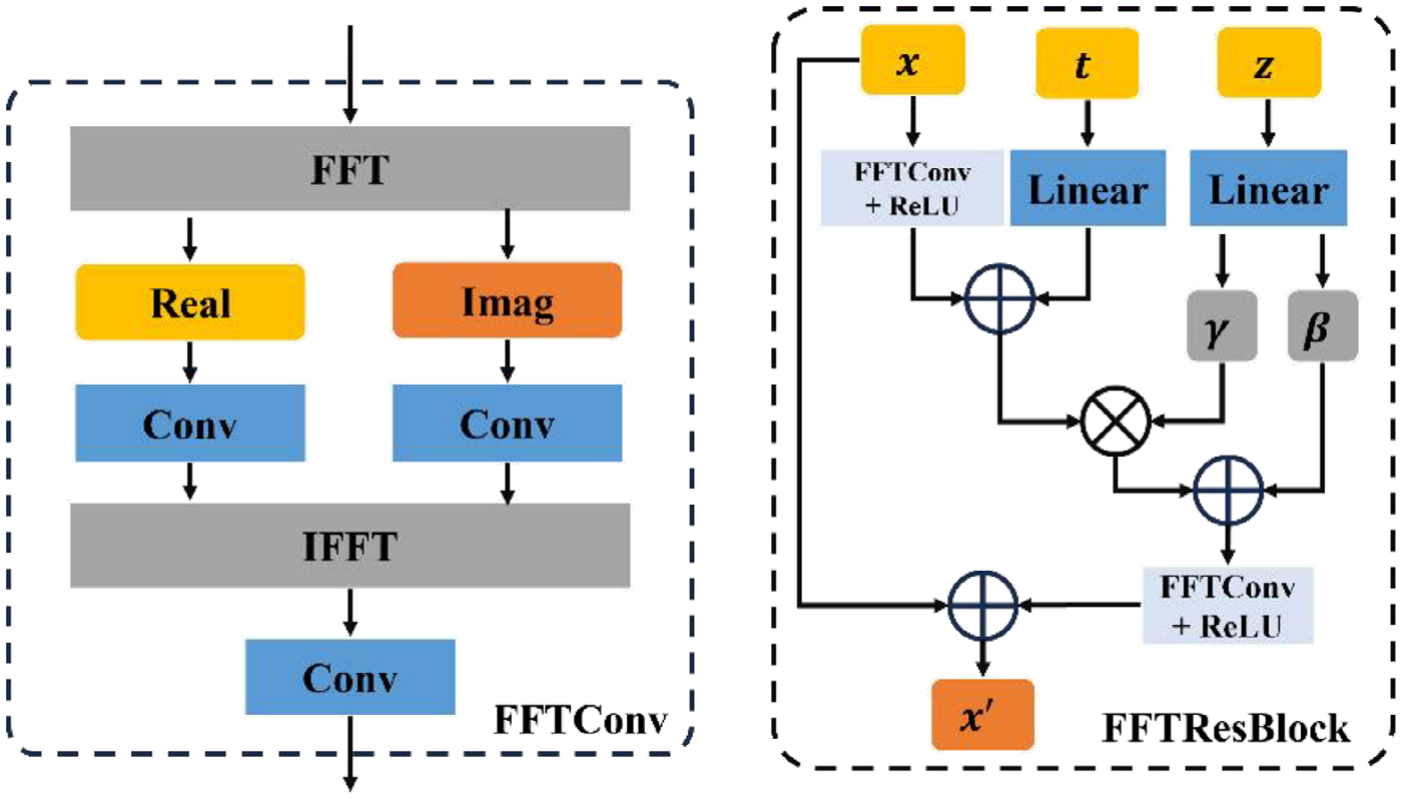
Architecture details of the proposed Fourier convolution modules.

By adding noise embedding to the features of the Fourier residual at each time step, we form a conditional Fourier residual block. The main difference between the conditional Fourier residual block and the Fourier residual lies in the additional embedding, which exists before the inverse transformation. Specifically, a time‐dependent noise embedding vector is generated and passed through a small MLP to produce scale and shift parameters. These parameters are used to modulate the frequency features via affine transformation before the IFFT step, allowing the model to adaptively adjust feature importance at different diffusion stages. This mechanism enables the conditional Fourier residual blocks to model dynamic transformations across the diffusion trajectory, enhancing the model's capacity to preserve fine‐grained frequency components critical for high‐fidelity MRI reconstruction.

### 2.4 Training strategy

The proposed model was trained on an RTX A6000 (48GB) GPU. We employed the Adam optimizer with a learning rate of 0.0002, and set the hyperparameters $\beta_{1}=0.5$ and $\beta_{2}=0.999$ to control the first and second moment estimates, respectively.

### 2.5 Experiment design

In this study, we collected the MRI of 22S‐duration imaging and full‐duration MRI of 31 fractional MRI scans from 14 patients. Among them, the MRI of 23 fractional scans were selected as the training set, and the MRI of 8 fractional scans were selected as the test set. The training set and test set are completely separated by patient identity, no overlapping patients exist across two subsets (23 patient fractional scans for training, 8 for testing). The predicted results on the test set were quantitatively and qualitatively compared with the state‐of‐the‐art methods to accurately evaluate the model's performance.

### 2.6 Evaluation metrics

In this study, four quantitative indicators, namely MAE (Mean Absolute Error), RMSE (Root Mean Squared Error), SSIM (Structural Similarity Index Measure), and PSNR (Peak Signal‐to‐Noise Ratio), were adopted to measure the performance of the MR reconstruction model on the test set. All four quantitative metrics (MAE/RMSE/PSNR/SSIM) are computed based on full 3D volumetric images instead of randomly selected single slices. Specifically, for the data of each case in the test set, the predicted results of MR reconstruction were compared with the fully imaged data, and the quantitative indicators were calculated in the following way.

MAE is a commonly used metric in statistics and machine learning to evaluate the performance of a predictive model. MAE measures the average magnitude of the errors between the predicted values and the actual values, without considering the direction of the errors. It is calculated by taking the average of the absolute differences between the predicted and actual values for a set of data points. Mathematically, if we have n data points, with predicted values $y_{pred,i}$ and actual values $y_{true,i}$, the MAE is calculated as follows:

(4) $$MAE=\frac{1}{n}\;\sum_{i=1}^{n}\left|y_{pred,i}-y_{true,i}\right|$$

A lower MAE value indicates a better performance of the model, as it means that the average difference between the predicted and actual values is small.

RMSE measures the square root of the average of the squared differences between the predicted values and the actual values. It takes into account both the magnitude and the direction of the errors. By squaring the differences, larger errors are given more weight than smaller ones, which makes RMSE more sensitive to outliers. Mathematically, for a set of (n) data points with predicted values ($y_{pred,i}$) and actual values ($y_{true,i}$), the RMSE is calculated as follows:

(5) $$RMSE=\sqrt{\frac{1}{n}\sum_{i=1}^{n}{\left(y_{pred,i}-y_{true,i}\right)}^{2}}\;$$

Similar to MAE, a lower RMSE value implies a better‐performing model. It provides a measure of the typical deviation of the predicted values from the actual values, and is often used in comparing different models or in evaluating the performance of a model over time.

PSNR is defined as the ratio of the maximum possible power of a signal to the power of the noise that affects the signal. The formula for calculating PSNR is as follows:

(6) $$PSNR=10log_{10}\left(\frac{I_{2}^{MAX}}{MSE}\right)$$

where $MAX_{I}$ is the maximum possible pixel value in the image, and MSE is the Mean Squared Error between the original image (I) and the reconstructed image (K), which is calculated as:

(7) $$MSE=\frac{1}{m\times n}\;\sum_{i=1}^{m}\sum_{j=1}^{n}{\left(I\left(i,j\right)-K\left(i,j\right)\right)}^{2}\;$$

where m and n are the dimensions of the image. A higher PSNR value indicates a better quality of the reconstructed or compressed image, meaning that the processed image is more similar to the original.

SSIM is a metric used to assess the similarity between two images, primarily focusing on the structural information of the images. Mathematically, the SSIM between two images I and K is calculated as follows:

(8) $$SSIM\left(x,y\right)=\frac{\left(2\mu_{I}\mu_{K}+C_{1}\right)\left(2\sigma_{IK}+C_{2}\right)}{\left({\mu_{I}}^{2}+{\mu_{K}}^{2}+C_{1}\right)\left({\sigma_{I}}^{2}+{\sigma_{K}}^{2}+C_{2}\right)}$$

where $\mu_{I}$ and $\mu_{K}$ are the means of images I and K respectively, $\sigma_{I}$ and $\sigma_{K}$ are the standard deviations of images I and K respectively, $\sigma_{IK}$ is the covariance between I and K, and $C_{1}$ and $C_{2}$ are small constants used to avoid instability in the calculation when the denominators are close to zero. The value of SSIM ranges from ‐1 to 1. A value of 1 indicates that the two images are identical, while a value of 0 indicates that the images are completely different. In general, the higher the SSIM value, the more similar the two images are in terms of their structural characteristics.

## 3 RESULTS

In this study, the state‐of‐the‐art paired image translation method, Refusion,^24^ and the unpaired image translation methods, CycleGAN^17^ and CSGAN,^25^ were selected as the comparative methods. The Refusion model uses the diffusion model as the basic architecture for paired image translation training. The CycleGAN model, a widely‐used unpaired image translation method, uses the generative adversarial network (GAN) as the basic architecture for unpaired image translation. Building upon CycleGAN model, CSGAN model employs the guidance of body mask and channel‐wise attention to further improve the unpaired image‐to‐image translation task.

Table 1 shows the quantitative comparison results between the proposed FSBD method and state‐of‐the‐art methods. The 22S‐duration baseline is used to quantify the discrepancy between the original low‐quality images captured under 22S‐duration imaging and high‐quality images acquired via full‐duration imaging. The FULL‐Fraction baseline represents the metric performance between two full‐quality standard MR images from different fractions of the same patient, which serves as clinical reference standard We note that this value is only used to provide the index fluctuation range derived from high‐quality complete images acquired in multiple fractions for the same patient, assisting in intuitively distinguishing image variations caused by the rapid reconstruction algorithm from inherent deformation deviations among different fractions. To ensure fair comparison, these image pairs of 22S‐duration and full‐duration MRI were first registered to correct for global positioning shifts before computing voxel‐wise metrics. After this preprocessing step, the reconstructed images generated by all MR reconstruction methods were spatially aligned to the full‐duration reference images, guaranteeing consistent spatial correspondence between the input data and ground‐truth labels across all methods.

**TABLE 1:** Quantitative comparison with state‐of‐the‐art methods.

	MAE↓	RMSE↓	PSNR↑	SSIM↑
22S‐duration	110.29 ± 21.37	169.16 ± 28.72	26.05 ± 1.83	0.871 ± 0.029
FULL‐Fraction	82.65 ± 27.81	176.44 ± 40.93	22.08 ± 2.28	0.746 ± 0.028
RefusionRefusion	99.95 ± 16.58	149.16 ± 24.08	26.77 ± 1.48	0.870 ± 0.031
CycleGAN	112.92 ± 24.17	172.55 ± 36.06	25.82 ± 1.84	0.881 ± 0.030
CSGAN	103.04 ± 20.76	162.29 ± 25.46	26.34 ± 1.88	0.878 ± 0.032
Our FSBD	86.00 ± 15.91	123.42 ± 24.46	28.44 ± 1.78	0.885 ± 0.030

Refusion utilizes the paired training strategy. The training data for the Refusion model consists of registered image pairs from two MRI sets (the 22S‐duration and full‐duration MRI), and each pair of images has a clear corresponding relationship. Since the registered image pair provides explicit supervision information, the Refusion model can directly learn the mapping from the source image (22S‐duration MRI) to the target image (full‐duration MRI). In comparison, CycleGAN, CSGAN and our FSBD adopt unpaired training to lower the dependency on perfectly matched image pairs.

As shown in Table 1, the Refusion model converged on the training set obtained MAE, RMSE, PSNR, and SSIM values of 99.95, 149.16, 26.77, and 0.870 respectively on the test set. Different from the paired training method, CycleGAN adopts an unpaired method for model training. The training dataset consists of two image sets (the 22S‐duration and full‐duration MRI), but there is no explicit correspondence between these images. Through unpaired training strategy, CycleGAN learns the latent mapping relationship between the two sets. The CycleGAN model converged on the training set obtained MAE, RMSE, PSNR, and SSIM values of 112.92, 172.55, 25.82, and 0.881 respectively on the test set, and the numerical indicators are inferior to those of the Refusion model. Based on the CycleGAN model, CSGAN model introduces the guidance of body mask to enhance the learning of unpaired image‐to‐image translation. The CSGAN model outperforms CycleGAN model by achieving the MAE, RMSE, PSNR, and SSIM values of 103.04, 162.29, 26.34, and 0.878 respectively.

The proposed FSBD model in this study employs the diffusion model as the basic architecture and adopts the unpaired training strategy. On the test set, FSBD obtained MAE, RMSE, PSNR, and SSIM values of 86.00, 123.42, 28.44, and 0.885 respectively. These results significantly outperform state‐of‐the‐art models (Refusion, CycleGAN and CSGAN). Above quantitative results effectively show that the proposed FSBD model, with the help of Schrödinger bridge diffusion model in Fourier frequency domain, can learn the best translation function from the 22S‐duration MRI set to the full MRI set among these state‐of‐the‐art methods, even in an unpaired training setting.

Figure 4 shows qualitative comparison of the visualization results of Refusion, CycleGAN, CSGAN and the proposed FSBD model. As illustrated in Figure 4, the CycleGAN and CSGAN model cannot reconstruct the internal anatomical structural details of the MRI, and many regions are incorrectly reconstructed as background regions. The Refusion model achieves better MRI reconstruction performance by restoring more detailed anatomical structure. However, it still struggles to produce consistent reconstruction results for internal structures. Nevertheless, the proposed FSBD model can accurately restore most of the internal anatomical structures. For example, for the zoom‐in organs shown in Figure 4, the CycleGAN, CSGAN and Refusion model fail to reconstruct the organ structure details. However, our proposed FSBD model accurately and consistently reconstructs the anatomical structure details. The error maps between the reconstructed MRI and full‐duration MRI are also shown in the Figure 4. Obviously, the error maps of internal organs generated by our FSBD model are the best, with closer mean error to zero, significantly smaller dispersion and fewer obvious outliers. These qualitative results further indicate the superiority of the proposed FSBD model.

**FIGURE 4:**
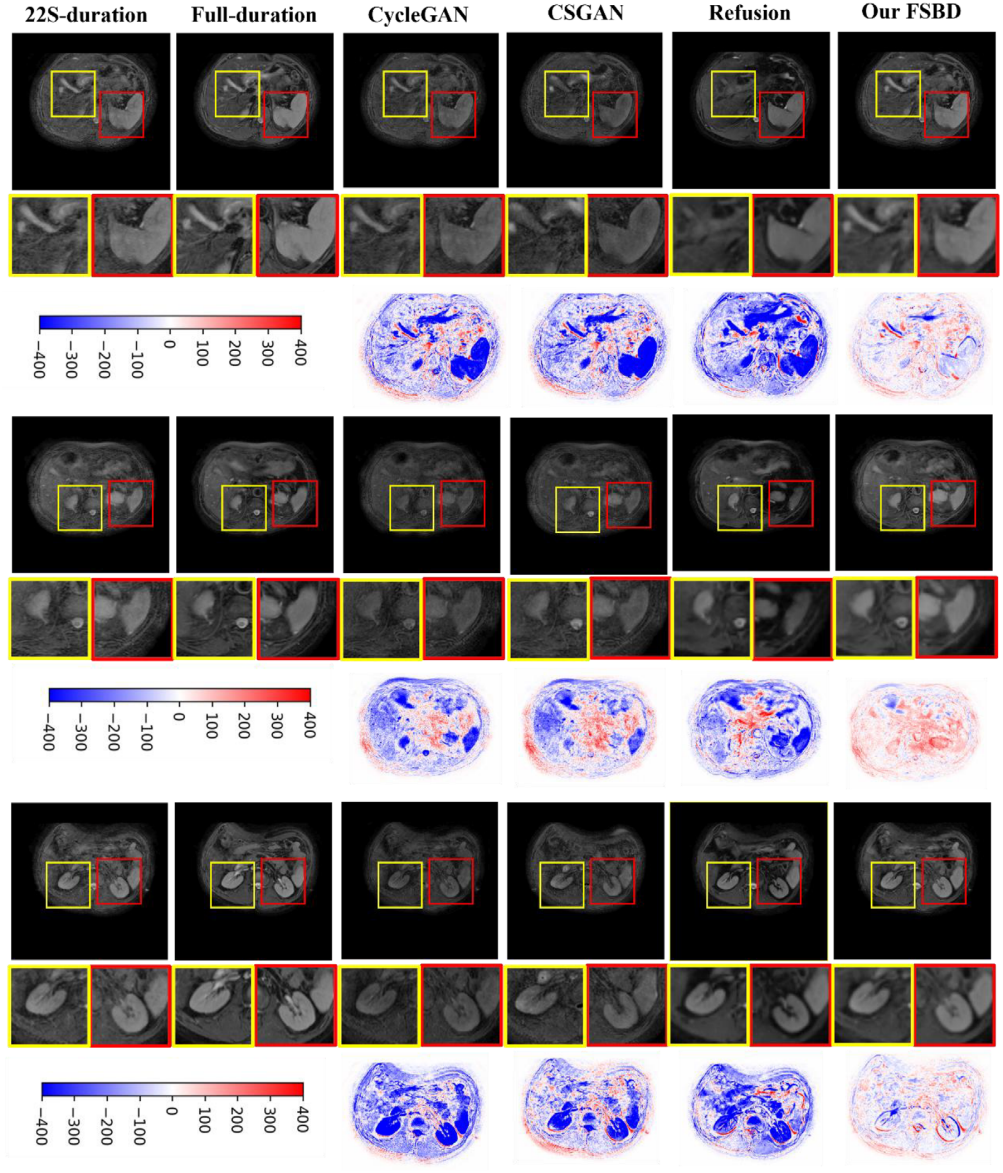
Visualized comparison between our FSBD model and state‐of‐the‐art methods (Blue‐red error maps correspond to absolute pixel‐wise MAE error between reconstructed and ground‐truth MRI.).

From above quantitative and qualitative comparison results, it is evident that the proposed model achieves significantly better MRI reconstruction results than existing state‐of‐the‐art methods, effectively demonstrating its superiority.

In terms of the inference speed, with the proposed FSBD model, accurate MR reconstruction results can be obtained in 20 s with the 22S‐duration MRI as input. The whole imaging time is about 42 s (22 seconds for MR imaging and 20 seconds for MR reconstruction). In this way, the whole processing time is significantly reduced from 3 min to less than one minute. As a result, the proposed method can significantly reduce scan time to enhance the patient experience, alleviating discomfort and physical fatigue for children, the elderly, and patients with claustrophobia. Additionally, it minimizes image artifacts caused by involuntary movements such as breathing and heartbeat, thereby reducing the need for repeat scans, improving examination efficiency, and optimizing clinical workflows.

## 4 DISCUSSION

Substituting the traditional T2‐weighted imaging which takes 360 s with the optimized T1‐weighted protocols lasting for only 120 s has effectively addressed the problem of rapid magnetic resonance (MR) imaging. Meanwhile, it ensures the image quality required for accurate contouring. These improvements not only increases patients' willingness to cooperate but also alleviates the anatomical changes that occur during the fractionated treatment. Additionally, this approach helps to reduce the burden on clinical resources, as indicated in references.^5^, ^6^, ^8^ This study presents the first implementation of sub‐one‐minute rapid magnetic resonance (MR) imaging (< 1 min) in an MR‐guided online adaptive radiotherapy (ART) workflow. This implementation achieves an over 80% reduction in scan time compared to standard MR sequences. This advancement renders the image acquisition phase of MR‐guided ART more time‐efficient than conventional cone‐beam computed tomography (CBCT) or CT‐guided ART systems, effectively addressing the long‐standing limitation of prolonged MR scan times in traditional MR‐guided radiotherapy.^4^, ^26^

Although specific optimizations for the Unity MR linear accelerator have not been reported, the successful application of related technologies in similar MRI reconstruction problems^10^, ^11^, ^12^, ^13^, ^14^, ^15^ indicates that U‐Net is well‐suited for single‐frame processing of MRI sequences, cascaded CNNs are suitable for processing dynamic sequences, and GAN‐based k‐space super‐resolution can serve as a future development direction. In contrast to previously reported rapid MR techniques, which primarily focuses on MR simulation or diagnostic applications,^26^ this work pioneers the integration of ultrafast MR imaging of Unity MR linear accelerator into online ART workflows. Although 22s undersampled and full‐length scans were acquired in the identical fraction, minor intra‐scan organ deformation still exists. Unpaired training helps the model learn robust anatomical transformation rather than simply memorizing pixel‐wise average mapping from paired data.

Methodologically, this study introduces a novel framework that combines Schrödinger Bridge theory with diffusion models to address the inherent challenges of ultrafast MR imaging, particularly noise amplification and resolution degradation caused by limited scan time. Compared to conventional denoising and image enhancement technique, such as compressed sensing, sparse reconstruction, and generative adversarial networks (GANs)^18^, ^19^, ^26^), the proposed model demonstrates obvious advantages. The diffusion model's iterative noise addition/removal process enables precise learning of MRI noise distributions. Schrödinger Bridge theory optimizes the transformation pathway via optimal transport constraints, ensuring stable denoising without mode collapse or over‐smoothing observed in traditional approaches.

The numerical metrics demonstrate that the proposed FSBD model achieves obviously better results than current Refusion, CycleGAN and CSGAN model. Concretely, the FSBD model achieves 86.00 MAE, 123.42 RMSE, 28.44 PSNR and 0.885 SSIM. The MAE and RMSE value measures the reconstruction accuracy. Our proposed FSBD model significantly reduces the reconstruction error of Refusion, resulting in evident reduction of 13.95 MAE and 25.74 RMSE. For the comparison with CycleGAN model, FSBD also achieves clear reduction of 26.92 MAE and 49.13 RMSE. Similar results can be observed for the comparison between the CSGAN and FSBD model. Moreover, the PSNR and SSIM value reflect the quality of reconstructed MRI. Notably, our FSBD model also achieves the highest reconstruction image quality, outperforming Refusion by 1.67 PSNR and 0.015 SSIM. Similar results can be observed for the comparison with CycleGAN. Compared to CycleGAN, the FSBD model obtains the enhancement of 2.62 PSNR and 0.004 SSIM, while surpassing CSGAN by 2.10 dB in PSNR and 0.007 in SSIM. These quantitative results effectively demonstrate the effectiveness of the proposed FSBD model. In addition, visualized comparison results clearly show the superiority of the FSBD model, by effectively reconstructing details of internal structures.

CycleGAN and CSGAN approximate the target distribution through the adversarial interplay between generators and discriminators, relying on adversarial loss functions. This makes their discriminators prone to overfitting or vanishing gradients, leading to mode collapse (repetitive generation of similar samples) and blurred details in the generated outputs. In contrast, the proposed FSBD method based on diffusion model iteratively model the data distribution via forward diffusion (gradually adding noise) and reverse denoising (learning the inverse process). Under the constraint of variational lower bound loss, the FSBD directly models the probability density of the data distribution, enabling more controllable generation processes that capture complex internal structures, such as textures and semantic consistency. As a result, FSBD achieves obviously better reconstruction accuracy, in terms of MAE and RMSE. The reconstruction error is largely decreased from the CycleGAN (112.92 MAE, 172.55 RMSE) and CSGAN (103.04 MAE and 162.29 RMSE), resulting in the MAE of 86.00 and RMSE of 123.42 for the proposed FSBD model. Visualized comparison shown in Figure 4 also clearly demonstrates the superiority of FSBD model by reconstructing the details of internal body tissue structures. Several existing works have explored DSBM and Fourier‐constrained diffusion models for general medical image reconstruction; distinct from these architectures, our FSBD embeds dedicated Fourier convolution block tailored for Unity abdominal accelerated MRI under unpaired training setting.

The Refusion model follows the “noise‐to‐data” paradigm of traditional diffusion model, progressively transforming data into pure noise before reconstruction. Its prior distribution offers limited information about the target, resulting in suboptimal generation guidance. Conversely, the proposed FSBD model implements a “data‐to‐data” process, leveraging prior knowledge directly from the target distribution via discriminative feature learning in frequency domain. Moreover, traditional diffusion models start from pure Gaussian noise, requiring numerous sampling steps and function evaluations to generate high‐quality samples, thus limiting efficiency. The Schrödinger bridge model, however, constructs a direct diffusion path between two distributions—for example, transitioning from a structured distribution (e.g., degraded images) to the target (clean images). By starting closer to the target distribution, the model maintains performance with fewer evaluations, achieving high‐quality generation with fewer steps and improved sampling efficiency. As a result, the proposed FSBD model effectively preserves finer internal structural details in generated images. Concretely, the MAE and RMSE are significantly reduced from 99.95 to 86.00, 149.16 to 123.42, respectively. As shown in the visualization results of Figure 4, MRI reconstruction result generated by the proposed FSBD model effectively retains the detailed anatomical structures, which is also validated by quantitative evaluation metrics.

Using the proposed FSBD model, a fast MRI reconstruction method can be achieved, with the reconstruction time of only 20 s. In this way, the overall MR imaging time is significantly reduced from 3 min to 42 s. A 1‐min MR imaging duration enhances patient comfort and compliance—mitigating discomfort, motion artifacts, and examination failures, particularly among vulnerable populations (pediatric, geriatric, pain‐afflicted, or claustrophobic patients)—while improving equipment throughput. As the gold standard for soft tissue contrast and target/Organ‐at‐Risk (OAR) delineation, MR's traditional 3‐min acquisition previously constrained its utility in real‐time applications. However, 1‐min imaging, synergized with AI‐powered image registration and rapid dose optimization (achievable within minutes), enables Online Adaptive Radiotherapy (Online ART) workflows—encompassing positioning, imaging, plan adaptation, and treatment—to be completed within the patient‐tolerable 20–30‐min window. When integrated with MR‐Linac systems, this advancement facilitates seamless “imaging‐adaptation‐treatment” integration, overcoming the 3‐min bottleneck that historically prolonged procedures and elevated risks of motion‐related inaccuracies or delays.

We acknowledge several limitations to be addressed in subsequent work: (1) The full‐duration standard scan and the 22‐s rapid scan in this study were acquired sequentially. Although patient motion was limited within 5 mm using abdominal belt fixation, subtle positional shifts and physiological organ deformation between fractions are inherent limitations of this work. Such displacements inevitably introduce biases to the quantitative comparison of the two sets of images. (2) Clinical patient data contain inherent biases including organ deformation and slight respiratory movement, which cannot be isolated. It is difficult to accurately distinguish whether image differences originate from reconstruction algorithm errors or inherent deviations caused by patient motion. (3) Global volumetric metrics were adopted for reconstruction evaluation in this study, which may conceal local errors in targets and organs at risk. We note that global averaged indices smooth local distortion, artifacts and detail loss and fail to reflect regional image quality for radiotherapy. (4) All data originate from abdominal sites, leaving other anatomical regions unverified. (5) The current experiment is a single‐center test, and future research is needed to investigate the performance of the proposed method on multi‐center dataset. (6) Current FSBD model requires extended training (nearly 2 weeks), which suggests that the reported results may not yet represent the method's full potential.

Planned improvements include: (1) Contour‐guided regional quantitative evaluation: Computing separate local metrics for the target and each OAR based on delineated contours to precisely evaluate anatomical reconstruction accuracy for online adaptive radiotherapy. (2) Dataset expansion and generalization: Enriching training samples and extending the framework to diverse sequences (T1/T2‐weighted, Balanced, DWI) across multiple anatomical sites. (3) Training acceleration: Investigating more effective diffusion mechanisms or lightweight architectures to reduce computational demands. (4) Integration with auto‐segmentation: Linking enhanced MRIs to automated organ delineation to streamline entire ART workflows.

## 5 CONCLUSION

We developed an online MRI‐guided radiotherapy rapid‐scan reconstruction method based on a Schrödinger bridge diffusion model for image enhancement. This approach improves the clinical efficiency of MRI‐guided radiotherapy while ensuring high‐quality fast‐scan MRIs, specifically designed for 1.5 T MRI‐based radiotherapy systems. It significantly reduces scan time from 3 min to 42 s (22 seconds for MR imaging and 20 seconds for MR reconstruction) while maintaining high‐resolution MR imaging, thereby ensuring precise radiation delivery and good patient compliance. This breakthrough establishes a technical foundation for precision online ART implementation.

## AUTHOR CONTRIBUTIONS

**Zhiqun Wang**: Conceptualization; methodology; investigation; formal analysis; writing—original draft. **Shaobin Wang**: Conceptualization; methodology; investigation; formal analysis; writing—original draft. **Nan Liu**: Investigation; data curation; writing—review & editing. **Qiu Guan**: Resources; validation; writing—review & editing. **Qi Chen**: Investigation; software; writing—review & editing. **Fang Chen**: Resources; data curation; writing—review & editing. **Bei Wang**: Investigation; visualization; writing—review & editing. **Yongguang Liang**: Investigation; formal analysis; writing—review & editing. **Xia Liu**: Investigation; validation; writing—review & editing. **Bo Yang**: Supervision; project administration; funding acquisition; writing—review & editing. **Lang Yu**: Supervision; project administration; conceptualization; funding acquisition; writing—review & editing. **Hongen Liao**: Supervision; project administration; conceptualization; funding acquisition; writing—review & editing. **Jie Qiu**: Supervision; project administration; conceptualization; methodology; funding acquisition; writing—review & editing.

## CONFLICT OF INTEREST STATEMENT

The authors declare no conflicts of interest.

## ETHICAL APPROVAL

This study was reviewed and approved by the Institutional Review Board of Peking Union Medical College Hospital (No. K4529).

## Data Availability

The data that support the findings of this study are available on request from the corresponding author. The data are not publicly available due to privacy and ethical restrictions.
